# Editorial: Intracranial pressure regulation in stroke

**DOI:** 10.3389/fstro.2023.1323696

**Published:** 2023-11-07

**Authors:** Daniel J. Beard, Lucy A. Murtha, Damian D. McLeod

**Affiliations:** ^1^School of Biomedical Sciences and Pharmacy, University of Newcastle, Newcastle, NSW, Australia; ^2^Heart and Stroke Research Program, Hunter Medical Research Institute, New Lambton Heights, NSW, Australia; ^3^School of Medicine and Health Sciences, Department of Cardiology, Carl von Ossietzky University of Oldenburg, Oldenburg, Germany

**Keywords:** acute ischemic stroke, intracranial hemorrhage, intracranial pressure elevation, collateral failure, stroke progression, early neurological deterioration (END)

Intracranial pressure (ICP) elevation occurs following both acute ischemic stroke and hemorrhagic stroke (intracranial hemorrhage), however, the mechanisms and role of transient ICP elevations have not been fully elucidated within the various stroke pathologies. Recent evidence suggests that ICP elevation may not only occur in malignant strokes but also in milder strokes and lead to decreases in perfusion to the brain resulting in infarct expansion and early neurological deterioration. Given the importance of ICP in influencing stroke outcome, it is imperative to elucidate the mechanisms of this rise in both ischemic and hemorrhagic stroke and develop novel non-invasive or minimally invasive techniques for measuring ICP in patients with mild strokes. In this Research Topic, we have assembled articles that highlight advancements in preclinical and clinical knowledge of ICP regulation in ischemic and hemorrhagic stroke pathophysiology, novel methods for long-term cerebrospinal fluid (CSF) sampling and clinical non-invasive ICP estimation.

Until recently, it was thought that ICP elevation was the result of large hemispheric infarction with large volumes of cerebral edema. However, the review by Hood et al. highlights recent studies demonstrating dramatic ICP elevation, independent of cerebral edema volume, in animal models of both large (middle cerebral artery occlusion, MCAo) and small ischemic stroke (photothrombotic stroke). Instead, ICP elevation after MCAo is accompanied by a significant increase in CSF outflow resistance after MCAo, which also compromises the perfusion of the ischemic penumbra by reducing cerebral perfusion pressure and the driving pressure across the leptomeningeal collateral vessels. Even an ICP elevation of 5 mmHg above pre-stroke levels can dramatically reduce the blood flow through the collateral-supplied “watershed” penetrating arterioles feeding the ischemic penumbra. These findings, coupled with human imaging studies indicating “collateral failure” as a likely mechanism of infarct expansion, suggest that ICP elevation is probably a dominant cause of “collateral failure” and early neurological deterioration (END) in ischemic stroke patients.

Likewise, intracerebral hemorrhage (ICH) is known to increase ICP, with previous dogma dictating that peri-hematoma edema leading to mass effect is the primary cause. However, the evidence for this is lacking. Although 60% of ICH studies have investigated edema as an endpoint, only 1% also measured ICP. To address this lack of data, Kalisvaart et al. conducted a retrospective analysis of ICH experiments in their lab. They found that measures of edema in the damaged hemisphere on their own were not predictive of average ICP response in the two ICH models investigated (collagenase and whole blood). Considering these findings, they propose future studies should assess mass effect and intracranial compliance (i.e., potential compensatory mechanisms such as reductions in tissue volume and CSF volume) in combination with long term ICP measurements, to provide a more complete picture of the pathophysiological response and patient outcome to ICH.

Given the emergence of changes in the CSF compartment as a mechanism of ICP elevation in ischemic stroke, it is vital to have optimal methods for long-term sampling of CSF and ICP measurement with minimal trauma to the underlying brain. Hao et al. present a novel method for cannulation implantation into the cisterna magna in rats, which would facilitate repeated CSF sampling and long-term monitoring of ICP. Using a modified cannula consisting of a puncture segment, connecting segment, fixing segment, and external segment, they were able to successfully cannulate the cisterna magna and attached the cannula to the skull to allow for animal recovery. They confirmed successful cannula placement physiologically with ICP waveform measurement as well as anatomically using CT imaging. The cannula remained patent for 7 days post-operatively in 77% of rats. Such a technique will be a potentially useful method for future investigations in the role of CSF in ICP elevation in stroke and other neurological diseases.

Although preclinical studies can give important insights into potential mechanisms of ICP, gaining similar insights in the clinic is far more difficult owing to the invasive nature of ICP monitoring in patients. Ophthalmic changes are promising non-invasive biomarkers of elevated ICP since they can be seen on clinical exam and ophthalmic imaging. Moss presents a mini review of the literature on retinal vein changes a potential biomarker for ICP elevation. They highlight that ICP elevation is known to increase retinal vein pressures through an increase in cerebral venous pressure, compression of venous outflow by elevated CSF pressure in the optic nerve sheath, and compression of venous outflow by optic nerve head swelling. In summary, the literature highlighted that retinal venous pressure can be measured using routine ophthalmic tests such as ophthalmodynamometry. However, although these tests have high clinical utility, there are still challenges in terms of the accuracy of a single clinical measurement to make a clinical diagnosis of elevated ICP, let alone provide a quantitative ICP value. A further complicating factor is the occurrence of optic nerve head swelling (papilledema) limiting the visualization of retinal vessels.

Our intention for this Research Topic is to highlight new and novel insights into the mechanisms of ICP elevation following stroke, present novel techniques for investigating these mechanisms and potential clinical tools to investigate these mechanisms in stroke patients.

## Author contributions

DB: Writing – original draft, Writing – review & editing. LM: Writing – review & editing. DM: Writing – review & editing.

